# Extrachromosomal microDNA Signature as a Candidate Biomarker in Pediatric Acute Lymphoblastic Leukemia

**DOI:** 10.1158/2767-9764.CRC-25-0419

**Published:** 2026-01-19

**Authors:** Ivan Brukner, Vincent Gagné, Alex Richard-St-Hilaire, Pascal Tremblay-Dauphinais, Claire Fuchs, Henrique Bittencourt, Teodor Veres, Daniel Sinnett, Maja Krajinovic

**Affiliations:** 1 Lady Davis Institute for Medical Research, McGill University, Montreal, Canada.; 2 CHU Sainte-Justine Research Center Azrieli, Charles-Bruneau Cancer Center, University of Montreal, Montreal, Canada.; 3 Department of Pediatrics, Faculty of Medicine, University of Montreal, Montreal, Canada.; 4 National Research Council Canada, Boucherville, Canada.; 5 Department of Pharmacology and Physiology, Faculty of Medicine, University of Montreal, Montreal, Canada.

## Abstract

Acute lymphoblastic leukemia (ALL) is the most common childhood cancer. Despite improved therapies, refractory and relapsed ALL remain the leading cause of cancer-related mortality in children. There is a need for accessible biomarkers for frequent, minimally invasive disease monitoring and prompt intervention. MicroDNA is a novel extrachromosomal DNA that preferentially originates from gene segments with high transcriptional activity and/or increased chromatin accessibility. We investigated whether microDNA-producing genes repertoire changes in a disease-dependent manner. We characterized microDNAs in 52 paired bone marrow (BM) and plasma samples from pediatric patients with ALL at diagnosis, relapse, and remission. No difference in the length or number of microDNA was noted across stages, but comparative analysis of microDNA profiles led to the identification of microDNA gene panels associated with active disease. The relative distribution of these genes was significantly different from that expected by chance (*P* < 0.0001). Analyses of BM samples identified a signature comprising 289 distinct microDNA-producing genes present in multiple patients at diagnosis and relapse but absent in remission. The best biomarker candidates were 11 microDNA-producing genes identified also in plasma samples at diagnosis and overrepresented in patients who relapsed (*P* = 0.006). MicroDNA from the same genes was confirmed in relapse plasma samples. All signature genes are known to be involved in cancer proliferation or drug response. MicroDNA seems to be a candidate for a novel class of biomarkers for ALL, with the potential to improve precision diagnostics, particularly through their identification in plasma samples. Further validation in an independent cohort of patients is warranted.

**Significance:**

Despite high cure rates, 10% to 15% of pediatric patients with ALL experience relapse. We identified a plasma-detectable microDNA signature from 11 genes that persists from diagnosis through relapse but disappears in remission. These findings demonstrate the potential of microDNA profiles as prognostic biomarkers in pediatric ALL, enabling noninvasive monitoring of disease status and risk stratification.

## Introduction

Acute lymphoblastic leukemia (ALL) is the most common childhood malignancy. Although cure rates have improved dramatically, 10% to 15% of patients still experience relapse during or after therapy and have poor prognosis (1, 2). Bone marrow (BM) biopsy is the gold standard for the diagnosis and monitoring of ALL; however, a single-site BM biopsy sample may not capture all malignant clones (3, 4). In contrast to biopsy samples, biomarkers that are easily accessible and can be sampled frequently—while reflecting full tumor heterogeneity—would enable less invasive disease monitoring throughout treatment (including early therapy phases and posttransplant follow-up). In this study, we explored the role of extrachromosomal circular DNAs (eccDNA) in ALL. EccDNAs vary in size from tens to millions of base pairs (bp) and contribute to morphologic and functional genomic diversity (5–7). Larger eccDNAs, also referred to as ecDNA (extrachromosomal DNA), often harbor oncogene or drug-resistant genes that drive tumor evolution (7, 8). Recently, a highly abundant subclass of small eccDNAs (<2 kb) was identified and termed microDNA (9). MicroDNAs are derived from unique, nonrepetitive genomic sequences and are enriched in gene-dense and regulatory regions. The distribution of gene regulation landmarks, such as gunanine-cytosine content and positioning near transcription start sites (TSS) or histone marks (7, 10, 11), suggests that microDNA primarily originates from active genes. Consistent with this, microDNA has shown lineage and cell-type specificity (9). Given their nonrandom genomic origins (notably near active genes), microDNAs may reflect specific cellular and gene expression states, indicating their potential utility as novel cancer biomarkers.

MicroDNAs are generated through DNA repair pathways or DNA damage induced by exogenous stimuli (e.g., chemotherapy; refs. 6, 10). Although microDNAs are too small to carry full protein-coding genes, Paulsen and colleagues (12) demonstrated that microDNAs can give rise to small functional regulatory RNAs (including microRNAs and microRNA-like siRNAs), suggesting that microDNAs could be transcribed and potentially regulate gene expression. EccDNAs can also trigger innate immune responses through the stimulator of the IFN gene pathway (13).

The potential use of microDNA in liquid biopsies is particularly attractive. Liquid biopsy analytes can be obtained using minimally invasive methods and allow real-time monitoring of tumor evolution (14, 15). MicroDNA molecules can escape into extracellular fluids and evade exonuclease degradation owing to their small size and circular structure. Indeed, microDNAs have been detected in the plasma and urine of healthy individuals, as well as in patients with cancer or kidney disease (16–18). These circulating microDNAs share key characteristics with tissue-derived microDNA (similar size profiles and preferential origins from active genomic regions), suggesting that the analysis of microDNA from liquid biopsy is feasible. Importantly, circular microDNAs have an inherent stability advantage over linear DNA as liquid biopsy biomarkers, and their circular structure makes them amenable to amplification via rolling-circle amplification. In this study, we conducted microDNA analyses in childhood ALL BM and plasma samples collected at different disease stages and investigated whether tumor-associated microDNA patterns could be leveraged for ALL diagnosis and prognosis.

## Materials and Methods

### Study samples

Fifty-two matched tumors (cell pellets from 2 mL BM) and plasma samples from patients with childhood ALL were obtained at diagnosis (tumor tissue), relapse (progressive disease), and remission (disease-free posttherapy samples). All samples were collected from the Childhood Cancer Biobank at Sainte-Justine University Health Center (SJUHC) in Montreal, Canada. Tumor tissue at diagnosis was available from 25 patients (21 with B-cell ALL and 4 with T-cell leukemia). Of these, 11 had relapse, and in 9 cases, material was available for analysis. Tissue from remission was available from 18 patients.

### MicroDNA extraction from nuclei

MicroDNA from BM samples was extracted from a cell pellet, as previously described (10), according to the HiSpeed Plasmid Purification Handbook (Qiagen). DNA was eluted in 1 mL of Tris-EDTA (TE) buffer and concentrated by adding 20 μg glycogen, 0.1 volume of 3 mol/L sodium acetate (pH 5.2), and 2 volumes of isopropanol, followed by centrifugation at 16,000 × *g* for 20 minutes. The pellet was resuspended in 20 μL 1 × TE buffer. DNA was then digested with exonuclease V (Epicentre Technologies) for 8 hours at a 1:5 dilution to remove linear double-stranded and single-stranded DNA and purified with a QIAGEN MinElute column (Qiagen).

### MicroDNA extraction from plasma

Plasma DNA was extracted according to the QIAamp Circulating Nucleic Acid protocol (Qiagen). To enhance the binding of nucleic acids to the QIAamp Mini membrane, 1 μg of poly(A) carrier RNA was added to each sample prior to extraction. The volumes of proteinase K and buffer were adjusted according to the plasma volume following the manufacturer’s instructions. The samples were processed on a custom vacuum manifold to achieve a recommended vacuum of −800 to −900 mbar (−24 to −26 in Hg). DNA was eluted in 45 μL of TE, and the entire eluate was vacuum-evaporated using Savant SpeedVac. The resultant pellet was resuspended in 20 μL of molecular-grade H_2_O. All DNA was digested with exonuclease V for 30 minutes at a 1:5 dilution to remove linear double-stranded and single-stranded DNA and purified with a QIAGEN MinElute column. A shorter exonuclease V digestion was used for plasma compared with BM to preserve DNA yield; prolonged digestion in these low-input samples led to overdigestion and sample loss in pilot tests.

### MicroDNA amplification

The eluted microDNA was used for rolling-circle amplification using degenerate 6-mer primers and Phi29 DNA polymerase. We used an in-house protocol using oligonucleotides containing internal C3 spacers to minimize the production of self-priming by-products (19, 20). Amplified microDNA concatemers were purified using Amicon Ultra-0.5 centrifugal filter devices (MilliporeSigma) and quantified using Qubit dsDNA BR Assay Kit (Invitrogen, Thermo Fisher Scientific, RRID: SCR_018095). Five hundred nanograms of amplified microDNA (500 ng) was fragmented using the Illumina DNA Prep kit to generate dual-indexed paired-end libraries following the Illumina DNA protocol with IDT for Illumina Unique Dual Indexes (set D, cat. #20027216).

### MicroDNA sequencing and bioinformatic and statistical analyses

Each sample’s microDNA was initially sequenced on an Illumina MiSeq platform (RRID: SCR_016379) using a 300-cycle MiSeq Micro v2 kit (∼3–4 million paired-end 2 × 150 bp reads per sample). In addition, to ensure adequate detection in low-yield samples, high-throughput sequencing was performed on an Illumina NextSeq 550 system (RRID: SCR_016381) using a 300-cycle mid-output kit (∼100–130 million paired-end 2 × 150 bp reads) for plasma samples obtained at relapse. Relapse plasma samples were sequenced at higher depth because initial standard coverage yielded few microDNAs; deeper sequencing increased detection sensitivity. Sequencing was performed at the Genomics Platform of the Institute for Research in Immunology and Cancer (RRID: SCR_027674), University of Montreal. Reads were mapped to the human hg19 reference genome using STAR (version 2.7.9a, RRID: SCR_004463), with the parameter chimSegmentMin = 20 to generate chimeric junction output and confirm the circular origin of microDNA clusters (10). Genomic regions matching microDNA coordinates were queried against the RefSeq database (RRID: SCR_003496) to annotate features such as 5′/3′ untranslated regions, intronic and exonic regions, and promoters (defined as 2 kb upstream of TSSs; ref. 10). The ENCODE Consortium data (RRID: SCR_015482; GM12878 lymphoblastoid cell line as a reference, RRID: CVCL_7526) were used to annotate the open chromatin regions [DNase I hypersensitive sites or Assay for Transposase-Accessible Chromatin using sequencing (ATAC-seq) peaks]. The coordinates of different annotations were downloaded from the UCSC server (RRID: SCR_005780). Previously published in-house R script (v4.0.5; ref. 10) was used for these analyses. Open chromatin sites were also annotated by intersecting microDNA loci with publicly available ATAC dataset from B-cell ALL (21).

We quantified the following for each sample: (i) the total number of unique microDNA fragments (normalized per million mapped reads), (ii) the total number of unique genes from which microDNA was produced (regardless of the number of fragments per gene), referred to as “microDNA-producing” genes, and (iii) the length distribution of microDNA fragments.

The expected “random” distribution of microDNA-producing genes was calculated based on a binomial model (using an online StatTrek calculator) given the number of individuals per stage (diagnosis, relapse, and end of treatment) and a 0.05 probability that microDNA is produced from a gene (based on the observed average of ∼2,000 microDNA-producing genes per individual of ∼40,000 total genes) and shared in ≥2 individuals per stage.

Because a single gene can produce many distinct microDNA fragments, each gene (and not each fragment) was considered as the statistical unit. Each gene contributed to one binary event per patient (detected vs. not detected). The gene candidate panel was defined through stepwise selection, first in BM and then in plasma, requiring a gene to produce microDNA in ≥2 diagnosis BM and in ≥2 plasma samples, to be absent in all remission BM and plasma samples, and to be confirmed present first in relapse BM and then in relapse plasma in ≥2 individuals.

Group comparisons between disease stages, or between observed versus expected distributions, were made using Mann–Whitney or *χ*^2^ tests as appropriate. Using gene-level approach, microDNA panel genes obtained from plasma samples at diagnosis were compared between patients who subsequently relapsed or not. Effect sizes are reported as ORs with 95% confidence intervals (CIs).

The mRNA expression levels of the identified microDNA signature genes were retrieved from available RNA-seq data for pediatric patients with ALL at our center (22). Gene expression (fragments per kilobase million) was quantified using Cufflinks (Ensembl v75 annotations) and compared between ALL cases and controls using the Mann–Whitney test. Expression data were available for 264 ALL samples, including 14 samples from which microDNA was extracted. In this latter subset, most samples were obtained at the time of relapse (9 of 14). Among the 264 patients, 260 had documented tissue origin and relapse status. Expression profiles were generated at diagnosis for 198 patients who did not later relapse, at diagnosis for 39 patients who subsequently relapsed, and at the time of relapse for 23 patients.

## Results

**Figure 1.**
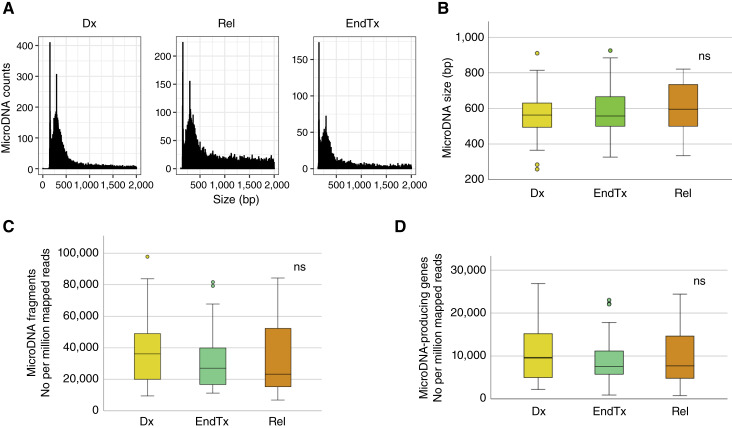
The lengths of microDNA obtained from BM at different stages of ALL development. **A,** MicroDNA counts relative to size distribution at diagnosis (Dx), relapse (Rel), and end of treatment (EndTx), with two peaks at ∼150 and 294 bp. **B,** Average microDNA size at different ALL stages with no difference [nonsignificant (ns) Mann–Whitney *P* > 0.05] observed between them. Average number (per million mapped reads) of microDNA fragments in **C** and microDNA-producing genes in **D** at different ALL stages with no significant difference (ns) observed between them. Dx, diagnosis; EndTx, end of treatment; Rel, relapse.

We profiled microDNAs in paired BM and plasma samples obtained from patients with childhood ALL at diagnosis (active disease, *n* = 25), relapse (progressive disease, *n* = 9), and remission (disease-free tissue at the end of treatment, *n* = 18). Analyses of microDNA from BM samples indicated that the average number of microDNAs per BM sample was 8,517. Of these, 2,178 were mapped to known genes (microDNA-producing genes). Individual microDNA fragments exhibited two modal lengths (∼150 bp and ∼294 bp; Fig. 1A) with a mean fragment length of 588 bp. Comparison of microDNA length distributions, total microDNA counts, and number of microDNA-producing genes (normalized per million reads) revealed no significant differences between the disease stages (Fig. 1B–D; all *P* > 0.05, Mann–Whitney).

**Figure 2.**
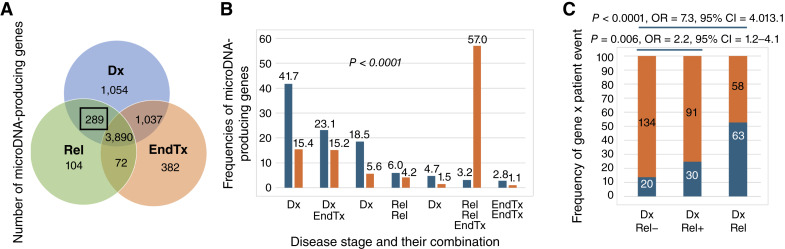
Prognostic value of microDNA in childhood ALL. **A,** Venn diagrams presenting the number of microDNA-producing genes at the different stages of ALL. Numbers indicate microDNA-producing genes obtained from BM in ≥2 individuals per stage, either unique to that stage or shared between stages. Of prognostic interest are microDNA-producing genes that are maintained from diagnosis to relapse but absent at the end of treatment (black box). **B,** Observed and predicted frequencies of microDNA-producing genes across Dx, Rel, EndTx, and their combinations. Observed (*orange bars*) represents the actual frequency of shared microDNA-producing genes detected in ≥2 individuals per stage. Predicted (*blue bars*) represents the expected frequency under a binomial model, assuming a 0.05 probability that microDNA is produced from a gene, (based on the observed average of ∼2,000 microDNA-producing genes per individual of ∼40,000 total genes) and shared in ≥2 individuals per group sizes of 25 Dx, 9 Rel, and 18 EndTx. Frequency is presented relative to the total number of microDNA identified in ≥2 individuals (6,828, sum of all numbers in Fig. 2A). The strong deviation (both higher-than-expected and lower-than-expected) between observed and predicted values (*χ*^2^*P* < 0.0001) indicates that these stage-specific patterns are nonrandom. **C,** Detection frequencies of an 11-gene microDNA signature in plasma at diagnosis and relapse. Each signature gene contributes one binary detection event per patient resulting in the total of 275 gene × patient opportunities (11 genes × 25 patients, 11 with subsequent relapse and 14 without). MicroDNA from the 11-gene panel detected 24.8% of gene × patient events in patients who later relapsed (DxRel+) compared with 13.0% in those who remained in remission (DxRel−). DxRel shows frequency of gene x patient events (52.1%) when they were analyzed from the plasma samples obtained both at diagnosis and relapse. Number and frequencies of gene x patient opportunities with positive and negative events (blue and orange section of the bar, respectively) with *P* values (one-tailed *χ*^2^) and OR with 95% CIs indicating differences between groups are indicated on the plots. Dx, diagnosis; EndTx, end of treatment; Rel, relapse.

We next analyzed how microDNA-producing genes (analyzed as gene-level binary events) from BM samples were shared between individuals and across disease stages. Table 1 summarizes the number of “non-shared” genes versus those present in ≥2 individuals per stage and Fig. 2A summarizes whether they are confined to one stage (unique to one stage) or shared between stages. The observed frequencies of shared and unique gene sets differed significantly from those expected by chance. (In Fig. 2B, “observed” refers to empirical gene counts and “predicted” to expected values under binomial distribution; *χ*^2^*P* < 0.0001).

**Table 1.** Number of microDNA-producing genes obtained from BM ALL samples.

​	Number of samples	Number of genes shared^a^ within the stage	Nonshared genes	Number of genes shared^a^ between stages		Total number of genes across all samples
				with Dx	with EndTx	
Dx	25	1,054	2,220	​	​	15,705
EndTx	18	382	1,426	1,037	​	13,370
Rel	9	104	1,154	289	72	11,386

Abbreviations: Dx, diagnosis; EndTx, end of treatment; Rel, relapse.

MicroDNAs are obtained at diagnosis, end of treatment, and relapse. Number of unique genes from which microDNA is produced and how they are shared between individuals of the same disease stage as well as between different disease stages.

a Sharing within group: the number of microDNA-producing genes shared between at least two individuals of the same stage. Sharing between groups: two individuals of each stage have the same microDNA-producing gene.

Analyses across ALL stages can reveal microDNAs arising from cancer-specific genes. MicroDNA-producing genes that are present in all three stages are likely specific to the BM environment, irrespective of malignancy, whereas cancer-related gene subsets are confined to diagnosis and relapse (Fig. 2A). Consistent with this, we defined two relevant subsets in our data: (i) a disease-specific signature – microDNA-producing genes present at diagnosis/relapse but lost in remission, and within this, (ii) a relapse-specific signature – a subset of microDNA-producing genes persisting from diagnosis through relapse. The relapse-specific signature from BM samples comprised 289 genes (highlighted by the black box in Fig. 2A), and their distribution among the samples obtained at diagnosis and relapse is presented in Supplementary Table S1. Next, we analyzed which of these 289 genes are also microDNA-producing genes in ≥2 plasma samples at diagnosis, while being absent in all remission plasma samples, leading to the identification of a candidate microDNA signature (or panel) composed of 11 genes. These 11 genes included regulators of apoptosis, signaling, and other cancer-related processes, as well as genes involved in drug response (Table 2).

**Table 2.** Gene composition of the microDNA signature.

Gene	Pathway/role
*AASS*	Amino acid metabolism and drug response
*ARFGAP1*	Cellular response to stimuli and regulator of mTORC1 signaling
*CASP7*	Apoptosis, linked to childhood leukemia
*KBTBD11*	Contains MYC-responsive element, linked to colorectal cancer risk
*LINC01134*	Cell viability and apoptosis
*TMEM52B*	Cancer cell survival and invasion
*DHFR*	Cell cycle, folate cycle, and drug response, linked to leukemia
*RHBDD2*	Membrane-bound proteases, linked to breast and colorectal cancer risk
*UBE2J2*	Apoptosis, linked to AML and drug response
*UCMA*	Cancer cell migration and invasion
*CYP2B6*	Metabolism and drug response, linked to leukemia

Abbreviations: *AASS,* aminoadipate-semialdehyde synthase; *ARFGAP1*, ARF GTPase-activating protein 1; *CASP7,* caspase 7;*CYP2B6*, cytochrome 450 2B6; *DHFR*, dihydrofolate reductase; *KBTBD11*, kelch repeat and BTB domain–containing 11; *LINC01134*, long intergenic non–protein-coding RNA 1134; *RHBDD2*, rhomboid domain–containing 2; *TMEM52B*, transmembrane protein 52B; *UBE2J2*, E2 ubiquitin–conjugating enzyme; *UCMA*, upper zone of growth plate and cartilage matrix associated.

Figure 2C uses a gene-level approach to analyze how the 11-gene microDNA signature derived from plasma of 25 patients at diagnosis differs between patients who subsequently relapsed and those who did not. Each signature gene contributes one binary detection event per patient, resulting in a total of 275 gene × patient opportunities (11 genes × 25 patients; 11 patients who later relapsed and 14 who did not). Using this approach, 11-microDNA gene panel detected 24.8% gene × patient events in patients who later relapsed (Fig. 2C, *DxRel+*) compared with 13.0% in those who did not (*DxRel*−; OR = 2.2; 95% CI, 1.2–4.1; *P* = 0.006). At the patient level, 63% of future-relapse patients versus 35% of nonrelapse patients had ≥2 signature genes detected at diagnosis.

**Figure 3.**
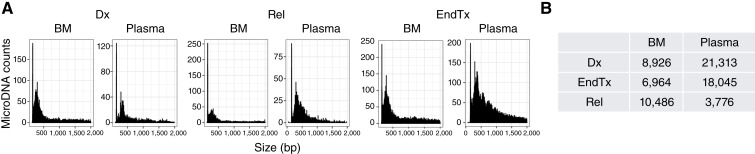
MicroDNA in BM and plasma samples at different disease stages of ALL. Length distribution in paired BM–plasma samples in **A**. Left, BM; right, plasma. Average number of microDNAs in BM and plasma samples in **B**. Dx, diagnosis; EndTx, end of treatment; Rel, relapse.

**Figure 4.**
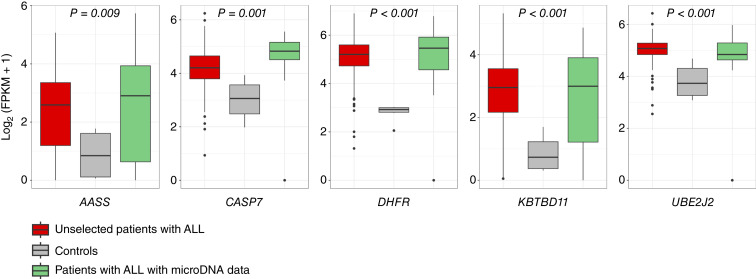
Normalized mRNA expression of microDNA signature genes (log_2_ FPKM + 1) from RNA sequencing data in ALL cohorts vs. controls. Box plots of gene expression levels derived from RNA-seq of 264 patients with ALL (in red), 6 controls (in gray), and 14 patients with ALL with microDNA data (in green). The expression is comparable between unselected patients with ALL and patients with ALL with microDNA data but significantly higher in comparison with the controls. The name of the genes and *P* value obtained by Mann–Whitney for the difference in expression between patients with ALL and controls is indicated on the plots. *AASS*, aminoadipate-semialdehyde synthase; *CASP7,* caspase 7; *DHFR*, dihydrofolate reductase; FPKM, fragments per kilobase of transcript per million mapped reads. *KBTBD11*, kelch repeat and BTB domain–containing 11; *UBE2J2*, E2 ubiquitin–conjugating enzyme.

Although the general length distribution of microDNA was similar between BM and plasma samples, the average number of microDNAs from plasma samples obtained at relapse was lower than that from BM and other disease stages (Fig. 3). This observation prompted higher coverage sequencing of relapse plasma samples. This additional sequencing confirmed that microDNA from each of the 11 signature genes was present in relapse samples in at least two individuals (Table 3), increasing the overall detection frequency of the signature genes to 52.1% (*P* < 0.0001, Fig. 2C). We also observed that all 11 genes produced multiple distinct microDNA fragments, indicating a gene-specific DNA instability (Table 3; Supplementary Fig. S1). MicroDNA fragments were derived from different regions of each gene, including the exons and promoter regions. Higher transcriptional activity at these loci was evidenced by the presence of open chromatin regions in reference to GM12878 lymphoblastoid cell line (LCL) and B-cell ALLs (Table 3), as well as by significantly higher gene expression relative to the controls (Fig. 4). The overlap with open chromatin regions in B-cell ALL samples was somewhat less frequent, which may reflect differences in the underlying cell populations but could also stem from methodologic differences in how chromatin accessibility was assessed in LCL versus B-cell ALL. Higher expression was observed in 5 of the 11 signature genes in the full cohort and in the samples analyzed for microDNA detection (Fig. 4). However, we did not observe expression differences between patients with and without relapse (Supplementary Fig. S2), suggesting that transcriptional activity alone is unlikely to account for relapse-associated variation in microDNA profiles.

**Table 3.** MicroDNA signature: Number and characteristics of microDNA per signature gene in plasma and BM at diagnosis and relapse.

Gene	Tissue	Individuals		Micro DNA	Exon	5′UTR/promoter	3′UTR	Intron	Open chromatin sites	
		Dx	Rel						LCLs	B-cell ALLs
*AASS*	P	5	5	42	8 (19)	2 (5)	1 (2)	31 (74)	16 (38)	5 (12)
	BM	2	2	4	1 (25)	0 (0)	0 (0)	3 (75)	3 (75)	2 (50)
*ARFGAP1*	P	3	4	20	11 (55)	3 (15)	0 (0)	6 (30)	20 (100)	10 (48)
	BM	2	3	5	5 (100)	0 (0)	0 (0)	0 (0)	5 (100)	2 (40)
*CASP7*	P	8	4	38	8 (21)	0 (0)	0 (0)	30 (79)	21 (55)	14 (24)
	BM	7	3	14	3 (21)	0 (0)	0 (0)	11 (79)	7 (50)	7 (50)
*KBTBD11*	P	5	4	39	1 (3)	34 (87)	4 (10)	0 (0)	35 (90)	14 (36)
	BM	2	2	5	0 (0)	4 (80)	1 (20)	0 (0)	5 (100)	2 (40)
*TMEM52B*	P	3	3	18	4 (22)	4 (22)	1 (6)	9 (50)	11 (61)	5 (28)
	BM	2	2	9	1 (11)	2 (22)	0 (0)	6 (67)	9 (100)	1 (11)
*DHFR*	P	4	2	30	2 (7)	0 (0)	27 (90)	1 (3)	2 (7)	1 (3)
	BM	2	2	5	0 (0)	0 (0)	0 (0)	5 (100)	1 (20)	0 (0)
*RHBDD2*	P	6	3	18	9 (50)	0 (0)	1 (6)	8 (44)	16 (89)	0 (0)
	BM	2	2	20	18 (90)	1 (5)	0 (0)	1 (5)	20 (100)	0 (0)
*UBE2J2*	P	5	6	32	8 (25)	1 (3)	1 (3)	22 (69)	22 (69)	2 (7)
	BM	2	2	8	5 (63)	0 (0)	0 (0)	3 (37)	8 (100)	0 (0)
*LINC01134*	P	5	4	79	28 (35)	0 (0)	0 (0)	51 (65)	61 (77)	15 (19)
	BM	2	2	9	1 (11)	0 (0)	0 (0)	8 (89)	5 (45)	3 (33)
*UCMA*	P	4	3	52	1 (2)	0 (0)	0 (0)	51 (98)	42 (81)	0 (0)
	BM	5	2	15	5 (33)	0 (0)	0 (0)	10 (67)	14 (93)	5 (33)
*CYP2B6*	P	2	3	8	1 (13)	0 (0)	1 (13)	6 (74)	4 (50)	0 (0)
	BM	2	2	4	0 (0)	0 (0)	0 (0)	4 (100)	2 (50)	0 (0)

Abbreviations: *AASS*, aminoadipate-semialdehyde synthase; *ARFGAP1*, ARF GTPase-activating protein 1; *CASP7,* caspase 7; *CYP2B6*, cytochrome 450 2B6; *DHFR*, dihydrofolate reductase; Dx, diagnosis; EndTx, end of treatment; *KBTBD11*, kelch repeat and BTB domain–containing 11; *LINC01134*, long intergenic non–protein-coding RNA 1134; P, plasma; Rel, relapse; *RHBDD2*, rhomboid domain–containing 2; *TMEM52B*, transmembrane protein 52B; *UB2J2*, E2 ubiquitin–conjugating enzyme; *UCMA*, upper zone of growth plate and cartilage matrix associated; UTR, untranslated region.

Number of samples with microDNA produced from each gene. Samples are obtained from (BM) or plasma at diagnosis or relapse. Several microDNAs originate from the same gene and are positioned in different gene regions. Number and frequencies of microDNA overlapping open chromatin sites identified as DNase I hypersensitive sites or ATAC-seq peaks in lymphoblastoid cell line (LCL GM12878) and using ATAC data from B-cell ALLs.

## Discussion

Comparative analyses of eccDNAs in various cancers have shown that tumor samples contain significantly greater numbers of microDNAs (and of larger size) than those found in healthy individuals (17, 23). In a previous *in vitro* study, we observed changes in microDNA count and size in LCLs after exposure to chemotherapy (10). However, in the present *in vivo* study, we did not observe such differences between leukemia BM samples and remission BM when analyzing microDNA from patient specimens.

Tumor-derived microDNAs have been detected in the plasma of patients with cancer, indicating that cancer cells can release specific microDNAs into circulation (16, 17). MicroDNA tends to originate near transcriptionally active sites and is enriched in open chromatin (9, 10). Thus, the detection of circulating microDNA might identify cancer-specific genes, providing a new biomarker for cancer using plasma or other liquid biopsy samples. Indeed, in this work, we have shown that microDNAs derived from a particular set of genes in both BM and plasma samples were specific for ALL and recurrent disease but were absent from remission samples. This observation is similar to findings by Luo and colleagues (23), who noted that the abundance of small eccDNAs originating from certain genes in the tissues and plasma of patients with solid tumors can have important diagnostic value (23). Similar observations have been reported for breast cancer, hepatocellular carcinoma, and lung cancer (24–26). In contrast, few data are available for hematologic malignancies. Analyses of ecDNA/eccDNA in acute myeloid leukemia (AML) and normal hematopoietic cells suggest that eccDNAs might play an important role in AML evolution (27). Specific patterns of eccDNA have also been found beyond cancer; distinctive profiles have been reported in patients with chronic kidney disease, diabetes, and systemic lupus erythematosus (18, 28, 29).

We provide evidence that extrachromosomal microDNAs can serve as informative biomarkers for pediatric ALL. The disease-specific microDNA signature comprised numerous sequences found across multiple patient diagnostic samples (BM and plasma) that were undetectable once the patients achieved remission, indicating an association with the presence of leukemia. Moreover, within this signature, we identified a specific microDNA gene subset associated with poor prognosis, which was maintained from diagnosis through relapse. To the best of our knowledge, this is the first report exploring role of microDNA in ALL. The identified microDNA signature spans multiple genes involved in leukemia biology, which makes it more robust to tumor evolution than a single mutation or clone-specific marker. Several microDNA sequences were traced to each gene locus, and the signature comprised 11 genes. All these genes have known roles in oncogenesis and treatment response (summarized in Table 2), reinforcing the biological relevance of the microDNA signature. The majority are involved in cancer cell survival and invasion, cell viability, and apoptosis and have been associated with breast cancer or colorectal cancer risk, as well as AML (30–37).

The identified signature includes dihydrofolate reductase (DHFR), an essential enzyme in the folate cycle and a major target of methotrexate (a key component of ALL treatment). Higher DHFR expression and polymorphisms in *DHFR* have been associated with a higher risk of relapse in ALL (38, 39). Cytochrome *P450 2B6* is well known for its role in drug metabolism; it also detoxifies many genotoxic xenobiotics, protecting cells from oxidative damage. Polymorphisms in this gene have been linked to leukemia risk (40). A similar association was found for polymorphisms in the gene coding for caspase 7 (*CASP7*; ref. 30). Aminoadipate–semialdehyde synthase (*AASS*) is involved in amino acid metabolism and can influence responses to asparaginase and corticosteroids (both used in ALL treatment); otherwise, altered AASS expression has also been seen in breast cancer (41). We also observed that all 11 genes produced multiple distinct microDNA fragments, indicating gene-specific DNA instability (Table 3; Supplementary Fig. S1). In paired diagnosis–relapse samples, microDNA counts for signature genes often increased or remained high at relapse, whereas these microDNAs were virtually absent in remission, bolstering their link to active disease. Gene expression analysis showed that 5 of the 11 signature genes were significantly overexpressed in ALL blasts at diagnosis relative to normal cells. For instance, *CASP7*, *AASS*, and *DHFR* were overexpressed in ALL cases versus controls, which might reflect cells primed for chemotherapy-induced stress (or, conversely, dysregulation of apoptosis pathways). Kelch repeat and BTB domain–containing 11 (which contains an MYC-responsive element) and E2 ubiquitin–conjugating enzyme were also highly expressed, consistent with their roles in growth signaling. Interestingly, certain genes in the microDNA signature are not classic players in ALL pathogenesis. Their inclusion may point to the general mechanisms of oncogenesis (such as epithelial–mesenchymal transition or microenvironment interactions) at play, even in leukemia. Further investigation is needed to clarify why these loci are prone to microDNA formation in patients with ALL. They may harbor fragile sites or repetitive elements that facilitate eccDNA generation or may represent hotspots of replication stress.

Our analysis focused on microDNA-producing genes that may be specific to ALL and its relapse. It is nevertheless important to note that stage-specific gene sets can show enrichment for pathways related to proliferation and signaling (diagnosis-specific, reflecting the initial leukemic state), posttherapy recovery or normal marrow activity (remission-specific), or resistance to treatment and apoptosis (relapse-specific).

Our study has several strengths that support confidence in the findings. First, the use of paired BM and plasma samples showed that plasma microDNAs mirror those in the marrow for a subset of tumor-associated microDNAs. Second, integration of transcriptome data provides mechanistic insight, indicating that the detected microDNAs are not random byproducts but are tied to leukemia cell biology.

Nonetheless, our study has some limitations. The candidate signature was derived and tested within a single-center cohort, and this same-cohort discovery/testing could inflate its performance. Future studies should validate the panel in larger independent populations to ensure generalizability. ALL is heterogeneous, and the current signature may be most relevant to the subtypes represented in our cohort. Future studies should examine microDNA patterns by ALL subtype to identify subtype-specific markers or confirm the broad applicability of the signature. Technical factors must also be considered: our relapse plasma samples received much deeper sequencing coverage than other samples, likely increasing microDNA detection in those cases; if remission plasma had been sequenced to similar depth, additional low-abundance microDNAs might have been detected. Similarly, we note that we used a shorter nuclease digestion for plasma DNA processing than for BM, to avoid sample loss; such protocol differences could affect microDNA yields and should be kept in mind when comparing across sample types. We did not have minimal residual disease (MRD)-positive samples to test whether the microDNAs from these samples could serve as an earlier indicator of relapse risk; this remains an intriguing question for future studies. Another future direction will be to investigate the cellular origin of these microDNAs. In majority of cases, the signature loci overlap known open chromatin regions, suggesting that these microDNAs likely originate from leukemia blasts. However, we cannot exclude the possibility that some microDNA is derived from nonmalignant BM niche cells or reflects leukemia-induced microenvironmental damage (42). The lack of xenograft or sorted cell experiments in our current study limits our ability to conclusively assign cellular origin; dedicated experiments (e.g., patient-derived xenografts or single-cell analyses) will be needed to disentangle tumor-versus-microenvironment sources of microDNA. Our finding of similar expression levels of signature genes in patients with and without relapse underscores that gene expression alone does not demonstrate microDNA generation; microDNAs more plausibly originate from loci characterized by high transcriptional activity and/or increased chromatin accessibility. Nevertheless, we cannot exclude the possibility that some signature genes are more disease-specific than relapse-specific.

In summary, we have demonstrated that extrachromosomal microDNAs have potential as candidate biomarkers for childhood ALL. Confirming the presence of leukemia-related microDNAs in patient plasma offers a new strategy for early relapse detection and personalized treatment monitoring. MicroDNAs represent a promising addition to other precision oncology approaches and merit further investigation and development. The stability of circulating microDNAs and their potential to capture disease-related genomic activity in plasma position them as valuable adjuncts to current minimal residual disease monitoring tools, including next-generation sequencing, which still has limited broad applicability due to its complexity and cost. Understanding microDNA biogenesis in leukemia might also reveal novel therapeutic targets for destabilizing cancer cell genomes.

## Supplementary Material

Supplemental Table 1 Supplemental Table 1: Contingency table of shared microDNA producing genes

Supplemental Figure 1 Supplemental Figure 1: Graphical presentation of the micro-DNA signature gene.

Supplemental Figure 2 Supplemental Figure 2: Normalized mRNA expression of microDNA signature genes (log2FPKM+1) as obtained from RNA-seq data in controls and ALL cohorts stratified by relapse.

## Data Availability

The in-house R script has been already published and made publicly available (10), and the data described in this study are uploaded and available in Sequence Read Archive at PRJNA1311073 (https://www.ncbi.nlm.nih.gov/sra/PRJNA1311073). Other data generated in this study are available upon request to the corresponding author.
