# Supplementary material for: Extrachromosomal microDNA Signature as a Candidate Biomarker in Pediatric Acute Lymphoblastic Leukemia
Source: Cancer Res Commun. 2026 Jan 19;6(1):143–51. doi: 10.1158/2767-9764.CRC-25-0419 (PMC12813666; doi:10.1158/2767-9764.CRC-25-0419)
Supplement: Supplemental Table 1 — Contingency table of shared microDNA producing genes [file crc-25-0419_supplemental_table_1_suppst1.docx]

**Supplemental Table 1.** **Contingency table of shared microDNA producing genes**

|  | | Number of Relapse Samples | | | |  |
| --- | --- | --- | --- | --- | --- | --- |
| Number of Dx Samples | Number of shared genes ^a^ | **2** | **3** | **4** | **5** | **Total** |
| 2 | Count | 94 | 20 | 5 | 0 | 119 |
| 3 |  | 52 | 18 | 1 | 1 | 72 |
| 4 |  | 26 | 8 | 6 | 0 | 40 |
| 5 |  | 21 | 7 | 2 | 0 | 30 |
| 6 |  | 10 | 6 | 0 | 0 | 16 |
| 7 |  | 8 | 1 | 1 | 0 | 10 |
| 8 |  | 1 | 0 | 0 | 0 | 1 |
| 9 |  | 2 | 0 | 0 | 0 | 2 |
| 10 |  | 0 | 0 | 0 | 0 | 0 |
| 11 |  | 0 | 0 | 0 | 0 | 0 |
| 12 |  | 0 | 0 | 0 | 1 | 1 |
| Total |  | 212 | 60 | 15 | 1 | **289** |

^a^microDNA derived for the same gene, shared between ≥ 2 samples obtained from bone marrow at diagnosis (dx) and relapse, but not present at the end of the treatment
